# Morphologic Changes in the Foveal Photoreceptor Layer before and after Laser Treatment in Acute and Chronic Central Serous Chorioretinopathy Documented in Spectral-Domain Optical Coherence Tomography

**DOI:** 10.1155/2013/361513

**Published:** 2013-09-19

**Authors:** Dominik Odrobina, Iwona Laudańska-Olszewska, Piotr Gozdek, Mariusz Maroszyński, Michael Amon

**Affiliations:** ^1^Ophthalmology Clinic of St. John Boni Fratres Lodziensis, Ulica Kosynierów Gdyńskich 61, 93-357 Łódź, Poland; ^2^Academic Teaching Hospital of St. John, Johannes von Gott Platz 1, 1020 Vienna, Austria

## Abstract

*Purpose*. To analyze microstructural changes in the external limiting membrane (ELM) and photoreceptor layer before and after early and late conventional laser treatment in central serous chorioretinopathy (CSC) in 12 months follow-up study. *Methods*. A retrospective observational study included Group A: 19 patients (19 eyes) with symptomatic acute CSC and Group B: 16 patients (16 eyes) with symptomatic chronic CSC. Retinal microstructural changes were analyzed with SD-OCT paying a particular role in examining the photoreceptor layer and ELM. *Results*. The length of the photoreceptors, prior to treatment, was approximately 84 **μ**m in Group A and 82,5 **μ**m in Group B. Twelve months after laser treatment, photoreceptor length was approximately 49 **μ**m in Group A and 43 **μ**m (range 20–55 **μ**m) in Group B. No patients in Group A had noticeable photoreceptor defects nor ELM defects, but in 15 eyes in Group B photoreceptor and ELM defects were detected (*P* < 0.0001). *Conclusions*. When analyzing the photoreceptor layer and ELM during active CSC, it is not possible to evaluate any irreversible changes which have already occurred in this layer. Damage to the photoreceptor layer and ELM in patients with chronic CSC was only found after laser treatment and the absorption of subretinal fluid.

## 1. Introduction 

Central serous chorioretinopathy (CSC) is a disease characterized by an idiopathic serous neurosensory retinal detachment secondary to leakage from the retinal pigment epithelium (RPE) [1]. From eighty to ninty percent of CSC regresses spontaneously over several months, but recurrence is observed in 33% to 50% of eyes [1, 2]. In spite of the many studies of CSC, there is continued controversy in the literature on the need, time, and method of treatment. 

 Due to rapid developments in modern imaging methods, it is possible to better understand changes in the retina and their influence on functional results after resolution of subretinal fluid in CSC. Spectral optical coherence tomography (SD-OCT) enables a highly detailed in vivo evaluation of the individual retina layers, especially the external limiting membrane (ELM) and the junction of inner and outer segments of photoreceptors [3, 4]. The development of SD-OCT technology in recent years has resulted in various authors noting that persistent leaks and subretinal fluid may cause RPE alterations and photoreceptor changes, which may correlate with worse visual acuity [5–7]. 

 The aim of the current study is to analyze the microstructural changes in the ELM and photoreceptor layer before and after early and late conventional laser treatment for CSC. 

## 2. Material and Methods

 A retrospective observational study included Group A, 19 patients (19 eyes) with symptomatic acute CSC (duration < 6 months) and Group B, 16 patients (16 eyes) with symptomatic chronic CSC (duration ≥ 6 months). 

 Treatment was carried out with a slit-lamp-mounted green light laser (IRIS GL Medical, Iridex Corp., Mountain View, CA, USA) using a fundus noncontact lens Volk 78. The parameters were spot size: 100–200 **µ**m, time: 0.1 s, and mean power used: 110 mW (70–140 mW) minimal grey retinal discoloration. The power to deliver burn in the leakage side was determined by test spots in the superotemporal quadrant. Between 1 and 3, laser spots were performed on the leakage side.

 Exclusion criteria were leakage side in the avascular zone <500 **µ**m from the fovea, previous laser treatment, PDT, TTT or anti-VEGF injection, any ocular diseases (vascular diseases, diabetic retinopathy, retinal detachment, ocular inflammation, etc.), or use of corticosteroids. 

 All patients were interviewed and underwent ophthalmologic examinations prior to treatment and 1, 2, and 12 months after laser treatment. Examinations included: BCVA using standard Snellen eye charts, intraocular pressure, anterior segment and fundus examination with Volk 78D and 90D lenses (Volk Optical Inc., Mentor, OH, USA), and SD-OCT analysis (Spectralis; Heidelberg Engineering, Heidelberg, Germany) with 3.9 **µ**m axial resolution and transverse resolution of 14 **µ**m. In each patient, we performed horizontal line scan through the fovea and made 19 B-scans on an area of 4.5 × 6 mm. In the experiment, we derived a medium value from three measurements performed for each patient. We compared the manual measurement performed by 2 ophthalmologists, and the results did not differ more than 5 **µ**m and were repeatable. We have taken an average of these two measurements. Additionally, fluorescein angiography was performed before laser treatment and 12 months after laser treatment.

 Photoreceptor length was measured as the distance between the external limiting membrane (ELM) and the most protruding outer segment of photoreceptors. Retinal thickness was measured as the distance between internal limiting membrane (ILM) and the most protruding outer segment of photoreceptors. The value of subretinal fluid in the centre of the fovea was also recorded. In SD-OCT, the fovea is recognized as the characteristic foveal depression where there is a lack of the following retinal layers: nerve fiber layer, ganglion cell layer, inner nuclear layer, and inner plexiform layer.

 Statistical analyses were carried out using Pearson product-moment correlation coefficient, Friedman's analysis of variance, Cochran's *Q* test, Fisher's exact test, Wilcoxon rank-sum test, and mixed-effects generalized linear models (GLMs). All analyses were conducted using Stata 12.1 Special Edition (StataCorp LP, College Station, Texas USA). The significance level was set to be *P* < 0.05. 

## 3. Results 

 In Group A, the mean age of the 19 patients was 36.7 ± 5.5 years. The mean age of the 16 patients in Group B was 48.8 ± 8.0 years. The mean visual acuity before laser treatment was 0.77 ± 0.16 in Group A and 0.51 ± 0.20 in Group B (*P* = 0.0007 Wilcoxon rank-sum test shows a statistically significant difference in visual acuity prior to treatment between Group A and Group B). The visual acuity was correlated significantly with the subretinal fluid, upon its resolution, in Group A only (*r* = −0.50, *P* = 0.0287 Pearson product-moment correlation coefficient); in Group B, the respective correlation was not substantial (*r* = −0.14, *P* = 0.6062). 

 Descriptive statistics on the following variables: length of the photoreceptors (the distance between external limiting membrane (ELM) and the most protruding outer segment of photoreceptors), retinal thickness (the distance between internal limiting membrane (ILM) and the most protruding outer segment of photoreceptors), and subretinal fluid in the centre of the fovea (measured before laser treatment 1 month and 12 months after laser treatment) are shown in Table 1. Hyperreflective subretinal deposits in 3 eyes in Group A and in 14 eyes in Group B demonstrated a noticeable bulge in the RPE before treatment (*P* < 0.0001 Fisher's exact test).

 During the first follow-up examination, one month after laser treatment, 16/19 eyes in Group A showed complete absorption of subretinal fluid whereas 11/16 eyes in Group B showed residual subretinal fluid (*P* = 0.004 Fisher's exact test). Two months after laser treatment, all eyes in Group A (19/19 eyes) showed complete absorption of subretinal fluid, whereas 2 eyes in Group B showed residual fluid (*P* = 0.19 Fisher's exact test). 

**Figure 1:**
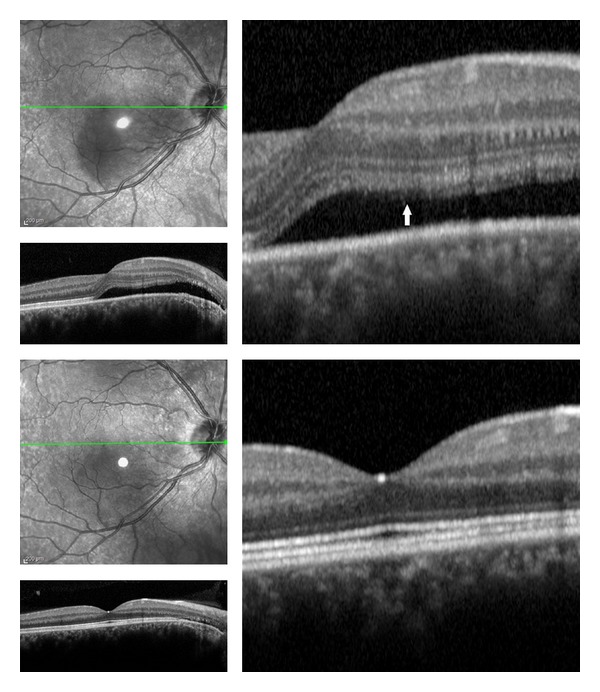
(Top) Acute central serous chorioretinopathy (CSC) before laser treatment. (Top right) Spectral optical coherence tomography (SD-OCT) shows elongation of the photoreceptors layer: distance before external limiting membrane (ELM) and most protruding outer segment of photoreceptors (white arrow); the RPE was flat. (Bottom) 12 months after laser treatment. (Bottom right) SD-OCT shows well-visible normal-thickness photoreceptor layer without any defects.

**Figure 2:**
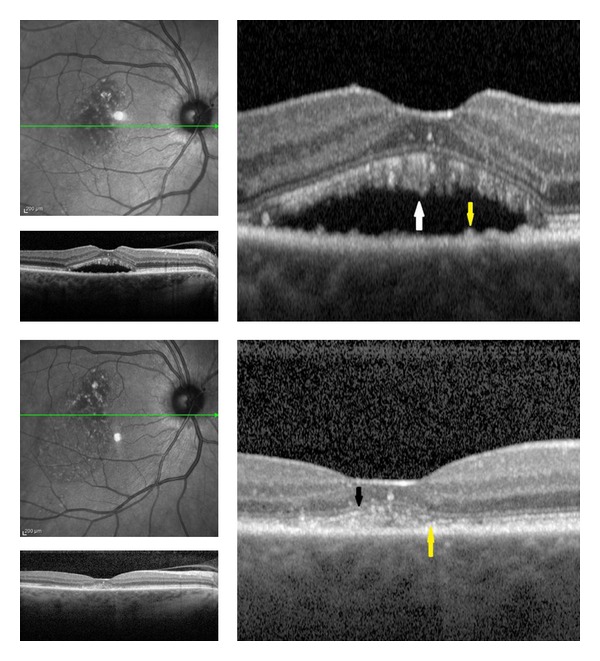
(Top) Chronic CSC before laser treatment. (Top right) SD-OCT shows elongation of photoreceptors layer with most protruding outer segment of photoreceptors (white arrow) and hyperreflective subretinal deposits demonstrated a noticeable bulge in the RPE (yellow arrow). (Bottom) 12 months after laser treatment. (Bottom right) SD-OCT shows disruption of the foveal photoreceptor layer and ELM (black arrow), and hyper-reflective subretinal deposits demonstrated a noticeable bulge in the RPE (yellow arrow).

 One month after laser treatment, hyper-reflective subretinal deposits in 3 eyes in Group A demonstrated a noticeable bulge in the RPE. However, after 12 months, the RPE was flat in all eyes (Figure 1). In Group B, hyper-reflective subretinal deposits demonstrated a noticeable bulge in the RPE in 14 eyes at 1 month after laser treatment which still persisted in all of those eyes after 12 months (*P* < 0.0001 Fisher's exact test) (Figure 2). 

 Twelve months after laser treatment, no patients in Group A had noticeable photoreceptor and ELM defects (Figure 1), but 15 eyes in Group B presented photoreceptor and ELM defects (*P* < 0.0001 Fisher's exact test) (Figure 2). 

 Twelve months after laser treatment, no patients in Group A had noticeable subretinal fluid, but 2 eyes in Group B showed residual fluid (*P* = 0.19 Fisher's exact test). The subretinal fluid absorption varied significantly over time (before laser treatment and one month and 12 months after laser treatment) in both investigated groups (*P* < 0.0001 in Group A, and *P* = 0.0001 in Group B, Cochran's *Q* test). However, the laser treatment was to be more effective, in the context of subretinal fluid absorption, in Group A in comparison to Group B (*P* = 0.0010 mixed-effects GLM). No recurrence was found in either Group A or B. No complications were detected in Group A. However, 12 months after laser treatment, CNV developed in 1 eye in Group B.

Changes in the length of the photoreceptors and subretinal fluid took place in a very similar pace and direction, both in Group A and in Group B (Table 1), except for the retinal thickness, where fluctuations were more distinct in Group A compared to Group B (*P* = 0.0132 mixed-effects GLM), and the visual acuity—a larger improvement was observed also in Group A versus Group B (*P* < 0.0001 mixed-effects GLM).

 At the final follow-up visit, 12 months after laser treatment, the mean visual acuity in Group A was 0.94 ± 0.09 and 0.71 ± 0.17 in Group B (*P* < 0.0001 Wilcoxon rank-sum test). There was a fitted model in which the visual acuity played the role of dependent variable, and repeated measures (before laser treatment and one month and 12 months after laser treatment), Group A versus Group B along with the length of the photoreceptors, subretinal fluid, and retinal thickness were set as the independent variables. Apart from the above-mentioned noticeable changes over a 12-month follow-up and between-group differences, the improvement of visual acuity was determined in both examined groups by appropriate retinal thickness (*P* = 0.0101 mixed-effects GLM) and observed diminution of the length of the photoreceptors (*P* = 0.0266 mixed-effects GLM). 

## 4. Discussion

 Analyzing and comparing the results from both groups of patients (acute and chronic CSC) prior to laser therapy did not reveal significant differences in the thickness of the retina, elevation of neurosensory retina, or microstructural changes of the retina. 

 During the 12 months of observation after laser treatment, subretinal fluid was noted to have been absorbed earlier in patients with acute CSC.

 When analyzing the photoreceptor layer in detail, defects were noted after laser treatment in 93% of eyes with chronic CSC but not noted in any case with acute CSC at the final examination. Defects in the photoreceptor layer conditioned visual acuity, which was statistically better after laser treatment in the group with acute CSC in comparison to the chronic CSC. Analysis of these results supports other authors, according to whom if subfoveal fluid persisted more than 4 months foveal atrophy may occur anytime [8], and degenerative changes often develop in the macula in CSC cases lasting longer than 6 months [9]; these changes correlate with visual acuity. We also observed that retinal thickness and subretinal fluid correlated significantly with visual acuity 12 months after treatment only with chronic CSC.

 Analyzing the length of the outer layer of photoreceptors before laser treatment in the acute and chronic CSC groups (no significant differences existed between these groups) showed its elongation in relation to healthy eyes, as previously reported by Matsumoto et al. [6]. 

 Damage to the photoreceptor layer in patients with chronic CSC was found only after laser treatment and absorption of subretinal fluid. It was not possible to evaluate any irreversible changes that had already occurred in the photoreceptor layer during active CSC. Elongation of the outer segments of photoreceptors may be due to a lack of photoreceptor phagocytosis by the RPE [6], while the duration of the elongation of this layer may affect the simultaneous initiation of photoreceptor apoptosis. The results also support Spaide [10], who analyzed the autofluorescence of CSC. Spaide hypothesized that increased autofluorescence may represent an photoreceptor outer segments phagocytized by the RPE. One month after laser treatment, only 3/18 eyes in the acute CSC group had hyper-reflective subretinal deposits (which were no longer present at the 12-month followup). Therefore, photoreceptor outer segment phagocytosis by RPE does not seem prevalent at the onset of CSC. However, subretinal precipitates, indicating phagocytosis, were found in 13/15 eyes in chronic CSC one month after laser treatment (and were still present at the 12-month followup). Furthermore, photoreceptor defects were found after laser treatment in 93% of eyes with chronic CSC indicating photoreceptor apoptosis. These facts suggest that both findings develop after the patients have had CSC for some time. Thus, a benefit of laser therapy for acute CSC may be that it limits the process of outer segment photoreceptor phagocytosis and apoptosis, which correlates with better visual acuity in the acute CSC patient group (*P* < 0.0001). 

## 5. Conclusions 

 In summary, microstructural changes of the retina shown in SD-OCT during active CSC show no statistically significant correlation to the duration of the disease. However, after laser treatment subretinal fluid absorption takes less time in patients with acute CSC. Photoreceptor and ELM defects were found in most patients with chronic CSC (*P* < 0.0001). Damage of the photoreceptor layer and ELM in patients with chronic CSC was found only after laser treatment and the absorption of subretinal fluid. An effect of these factors is that early laser treatment for CSC results in better visual acuity than late laser treatment (*P* < 0.0001). Early laser treatment may cause early resolution of CSC, which may prevent situations in which about 10%–20% of eyes did not heal spontaneously [1, 2]. Early treatment may also prevent photoreceptor damage and decreased visual acuity. 

## Figures and Tables

**Table 1:** Computed descriptive statistical parameters for length of the photoreceptors (**µ**m), retinal thickness (**µ**m), and subretinal fluid (**µ**m) in the examined eyes with symptomatic acute CSC versus chronic CSC—before treatment and 1 month and 12 months after laser treatment.

	Before laser treatment				1 month after laser treatment				12 months after laser treatment				*P* ^ a^
	Mean	SD	Min.	Max.	Mean	SD	Min.	Max.	Mean	SD	Min.	Max.	
	Symptomatic Acute CSC												
(1) Length of the photoreceptors (*µ*m)	84.68	20.66	59	125	59.21	21.25	36	122	49.32	15.46	31	93	<0.0001
(2) Retinal thickness (*µ*m)	218.00	48.21	147	303	208.16	41.23	134	270	228.74	15.36	201	248	0.0200
(3) Subretinal fluid in the centre of the fovea (*µ*m)	221.58	101.46	71	434	8.21	16.62	0	60	0.00	0.00	0	0	<0.0001

	Symptomatic Chronic CSC												
(1) Length of the photoreceptors (*µ*m)	82.50	23.09	26	123	56.81	18.41	20	90	42.88	8.85	20	55	<0.0001
(2) Retinal thickness (*µ*m)	188.88	57.55	64	337	173.88	47.86	65	260	194.94	54.78	65	260	0.1300
(3) Subretinal fluid in the centre of the fovea (*µ*m)	199.19	87.24	37	315	27.06	30.31	0	111	12.19	30.08	0	111	<0.0001

*P* ^ b^	(1) 0.8554	(2) 0.0944		(3) 0.6911	(1) 0.9077	(2) 0.0446		(3) 0.0145	(1) 0.6067	(2) 0.0218		(3) 0.0520	

^a^ *P* values provided for any intragroup comparison, considering changes over time (Friedman's ANOVA was performed).

^b^ *P* values provided for any between-group (unpaired) comparison (Wilcoxon rank-sum test was carried out).
